# Efficacy and cost-effectiveness of a community-based model of care for older patients with complex needs: a study protocol for a multicentre randomised controlled trial using a stepped wedge cluster design

**DOI:** 10.1186/s13063-018-3038-0

**Published:** 2018-12-04

**Authors:** Irina Kinchin, Susan Jacups, Jennifer Mann, Rachel Quigley, Desley Harvey, Christopher M. Doran, Edward Strivens

**Affiliations:** 1 Centre for Indigenous Health Equity Research and School of Health, Medical and Applied Sciences, CQUniversity, Cairns, QLD Australia; 2 The Cairns Institute, James Cook University, Cairns, QLD Australia; 3 Medical Services, Torres and Cape Hospital and Health Service, Cairns, QLD Australia; 4 Cairns and Hinterland Hospital and Health Service, Cairns, QLD Australia; 5 College of Medicine and Dentistry, James Cook University, Cairns, QLD Australia; 6 Division of Tropical Health and Medicine, James Cook University, Cairns, QLD Australia

**Keywords:** Model of care, Comprehensive geriatric assessment, Integrated care, Effectiveness, Cost utility analysis, OPEN ARCH, Complex care needs, Elderly, Quality of life

## Abstract

**Background:**

Community-dwelling older persons with complex care needs may deteriorate rapidly and require hospitalisation if they receive inadequate support for their conditions in the community.

**Intervention:**

A comprehensive, multidimensional geriatric assessment with care coordination was performed in a community setting—Older Persons ENablement And Rehabilitation for Complex Health conditions (OPEN ARCH).

**Objectives:**

This study will assess the acceptability and determine the impact of the OPEN ARCH intervention on the health and quality of life outcomes, health and social services utilisation of older people with multiple chronic conditions and emerging complex care needs. An economic evaluation will determine whether OPEN ARCH is cost-effective when compared to the standard care.

**Methods/design:**

This multicentre randomised controlled trial uses a stepped wedge cluster design with repeated cross-sectional samples. General practitioners (GPs; *n* ≥ 10) will be randomised as ‘clusters’ at baseline using simple randomisation. Each GP cluster will recruit 10–12 participants. Data will be collected on each participant at 3-month intervals (− 3, 0, 3, 6 and 9 months). The primary outcome is health and social service utilisation as measured by Emergency Department presentations, hospital admissions, in-patient bed days, allied health and community support services. Secondary outcomes include functional status, quality of life and participants’ satisfaction. Cost-effectiveness of the intervention will be assessed as the change to cost outcomes, including the cost of implementing the intervention and subsequent use of services, and the change to health benefits represented by quality adjusted life years.

**Discussion:**

The results will have direct implications for the design and wider implementation of this new model of care for community-dwelling older persons with complex care needs. Additionally, it will contribute to the evidence base on acceptability, efficacy and cost-effectiveness of the intervention for this high-risk group of older people.

**Trial registration:**

Australian New Zealand Clinical Trials Registry, ACTRN12617000198325p. Registered on 6 February 2017.

**Electronic supplementary material:**

The online version of this article (10.1186/s13063-018-3038-0) contains supplementary material, which is available to authorized users.

## Background

The Australian healthcare system experiences significant demand in health service utilisation due to an aging population coupled with more complex health conditions [1]. Complex health conditions are the leading cause of illness, disability and death in Australia, accounting for 90% of all deaths in 2011 [1]. Hospitalisation for many complex conditions is preventable, yet they remain the most common hospital admission category [1].

Community-dwelling older patients with complex health needs may deteriorate rapidly and face hospitalisation if their conditions are poorly managed or poorly coordinated in the community. Once hospitalised, these often frail individuals may face excessively long hospital stays, which in turn increase their risk of hospital-acquired infections, delirium, falls and cognitive decline [2]. They become more dependent and may require permanent placement in a residential aged care facility, rather than returning home as a self-caring individual. It is not unusual for such patients to be hospitalised without great acute need.

Strong evidence suggests that older people will have better health outcomes if appropriate preventative care is provided in the community and early in the trajectory of the person’s illness [2]. In 2009 the National Health and Hospital Reform Commission (the Commission) recommended major changes to the health service delivery for patients with complex needs [3]. Of central interest are older people with interrelated medical, functional and psychosocial issues, increased risk of functional deterioration and unplanned institutional care. The Commission reinforced the task to expand specialist services in the community to address the needs of older people [3]. Several such models of care have been developed. In comprehensive geriatric assessment, such as the geriatric evaluation and management (GEM) model of care, an interdisciplinary team of healthcare professionals assesses an older person’s medical, functional, psychosocial, nutritional and environmental needs [4]. This interdisciplinary team creates a comprehensive plan of care that is communicated to the person’s physician. The model demonstrats a multidisciplinary, coordinated solution designed to facilitate care transition and improve health outcomes for older people with multidimensional health needs [4].

Published programmes utilising the GEM in Australia have shown promising results, including the Victorian Hospital Admission Risk Program, which reported 35% fewer Emergency Department (ED) attendances, 52% fewer admissions and 41% fewer days in hospital [5]. Similarly, the Reengineered Hospital Discharge Program [6], Better Outcomes for Older Adults Through Safe Transitions and State Action on Avoidable Re-hospitalisations programmes have shown improvements in reducing hospital readmissions and hospital utilisation such as length of stay [7]. The Geri-FITT model of care which involves a ‘floating interdisciplinary geriatric team’ to provide continuity of care through developing and facilitating transitional care plans and educating patients and care providers about geriatric care principles has demonstrated improved quality care transitions and greater patient satisfaction [8].The most similar study to the one proposed here was the ‘Patients First Model of Care’, which targeted people over 55 years old who frequently presented to the ED or were at risk of presenting to the ED. This study involved a comprehensive needs assessment by a community-based care facilitator, with care coordination and facilitation of access to a suite of services including allied health therapies where required. Findings from this community coordinated study reported a 27.9% reduction in hospital admissions and a 19.2% reduction in bed days without increasing overall costs to the health system [9].

Recent research on care transitions of older people across acute, sub-acute and primary care identified underutilisation of the GEM model in a community setting in north-eastern Australia [4]. In this region, an integrated model of comprehensive geriatric assessment, care coordination and rehabilitation is currently only available for hospital in-patients, with no preventative services available to prolong independent community living. Furthermore, sub-acute services for community-dwelling older persons are found to be fragmented; the access to health pathways is unclear, potentially leading to a perverse incentive for hospital admissions to access in-patient geriatric services.

This trial aims to address the gap by delivering and evaluating a comprehensive, multidimensional geriatric assessment with care coordination in a community setting. The Older Persons ENablement And Rehabilitation for Complex Health conditions (OPEN ARCH) model of care evolved from an initial study that explored patient well-being post hospitalisation [10]. That completed study examined patients’ transition back to primary healthcare and their access to allied health and social support services within the community [10]. This current OPEN ARCH intervention builds on previous research [4, 10] and extends the service to preventative service delivered in a community setting; it creates a direct path from a general practitioner (GP) to a community-based geriatrician-led Healthy Ageing Clinic for comprehensive interdisciplinary assessment and care planning. The OPEN ARCH intervention will facilitate timely access to primary healthcare, including allied health services and social supports within the community. It is envisaged that the OPEN ARCH intervention will delay health decline by increasing individual capacity, when supported by a team of community healthcare professionals, who monitor and manage the community-dwelling complex older patients within the community. Such coordinated care support is expected to reduce the likelihood of the aforementioned cascade of events that leads to hospitalisation, and eventual loss of independence following hospital discharge.

## Objectives

The objective of the study is to assess the acceptability of the OPEN ARCH intervention and its impact on the health and quality of life outcomes, health and social services utilisation of older people with multiple chronic conditions and emerging complex care needs. An economic evaluation will determine whether OPEN ARCH is cost-effective when compared to the standard care. In particular, this study will address the following research questions:

- Does the OPEN ARCH intervention result in reduced hospital care demand as measured by Emergency Department (ED) presentations, hospital admissions and in-patient bed days?
- Does the OPEN ARCH intervention result in reduced functional decline and dependency, and enable patients to live in the community for longer?
- Does the OPEN ARCH intervention result in an improved quality of life for participants?
- Is the OPEN ARCH intervention considered by participants to be an acceptable model of care?
- Is the OPEN ARCH intervention a cost-effective means to achieving better health and quality of life outcomes for at-risk older persons when compared to standard care?

## Methods

**Fig. 1:**
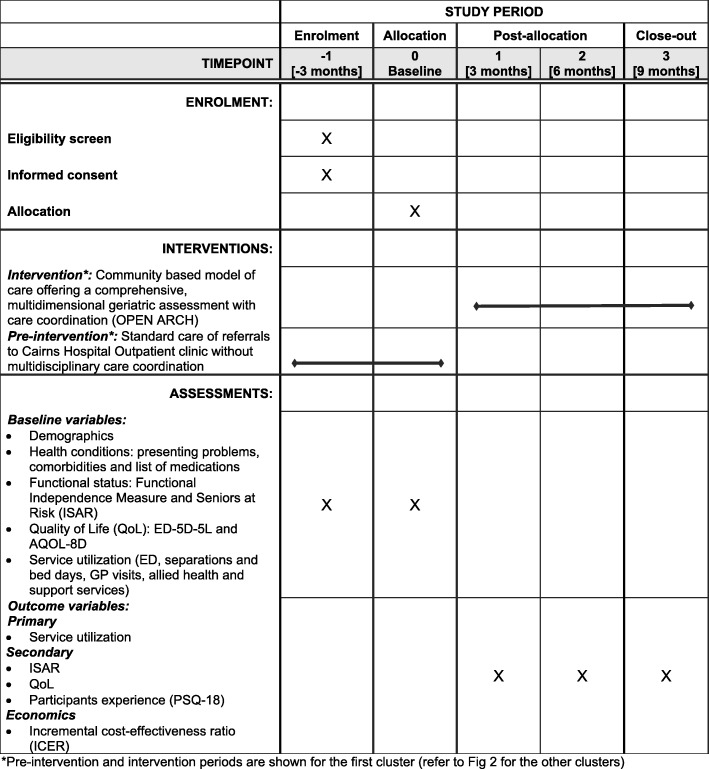
Standard Protocol Items: Recommendations for Interventional Trials (SPIRIT) figure of the Older Persons ENablement And Rehabilitation for Complex Health conditions (OPEN ARCH) intervention

The items in this study protocol comply with the Standard Protocol Items: Recommendations for Interventional Trials (SPIRIT) checklist (see the SPIRIT Checklist and figure in Additional file 1 and Fig. 1) [11, 12]. Adherence to SPIRIT allows transparency and completeness of the trial protocol for the benefit of investigators, trial participants, research ethics committees and other key stakeholders [11].

### Study setting

The study will be conducted at two sites in Far North Queensland (Australia): Cairns and Kuranda. Cairns is a regional centre on the north-east coast of Australia with a population of 160,285, of whom 11.6% are over the age of 65 years [13]. Kuranda is a rural community adjacent to Cairns in the north-west with a population of 4684, of whom 16.4% are over the age of 65 years [14].

### Trial design

**Fig. 2:**
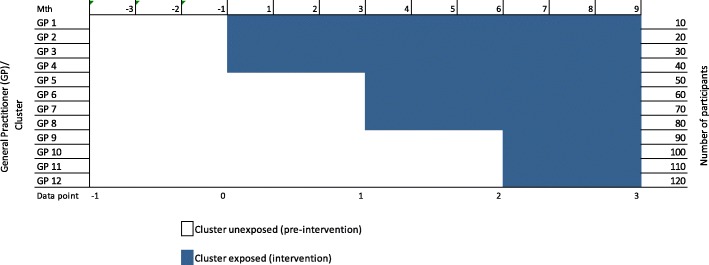
Schematic of the study design. Adapted from Hemming et al. [38]

A multicentre, randomised controlled trial using a stepped wedge cluster design with repeated cross-sectional samples will be utilised to measure participants’ acceptability of the model, health outcomes, services utilisation and economic viability of the OPEN ARCH intervention (see Fig. 2).

The stepped wedge design allows all participants to receive the intervention, which is the preferred methodology for studies where the intervention is predicted to do more good than harm [15]. It allows patients to act as their own controls and detect trends and changes associated with time [15, 16].

### Recruitment of general practitioners

The OPEN ARCH intervention builds on a successful collaboration between Cairns and Hinterland and Hospital and Health Service clinicians with local GP practices. Individual GPs with an interest in geriatrics will be approached via clinical and professional networks to participate in the study. A minimum of 10 GPs within the Cairns area (urban) and the Cairns Hinterland region (rural) will be approached to participate in the study. The project manager will conduct informed consent for the GP to participate.

### Randomisation of general practitioners

All recruited GPs will be randomised as ‘clusters’ at baseline using simple randomisation [17]. Such cluster randomisation allows control for the ‘between GPs variance’. A minimum of two clusters (two GPs) will be commencing the intervention at each step, as per the recommendations of Barker et al. [16], to ensure that the intervention effect estimator maintains the nominal 5% significance level and is reasonably unbiased. All clusters will ultimately receive the intervention for the same length of time.

### Staffing

For the purposes of this study, the following staffing will be recruited: a project manager, who will facilitate GP engagement, patient recruitment and consent, data collection, data management and reporting; a community-embedded specialist in geriatric medicine to provide comprehensive geriatric assessments and management plans at the GPs’ rooms; and two community-based care coordinators (enablement officers), who will facilitate access to healthcare services, social supports and self-management options. These positions are solely community embedded, designed to coordinate hospital-based specialist geriatric and other primary care services, such as social care services, allied health and rehabilitation services.

### Participants’ eligibility criteria

#### Sample size

Bird et al. [5] reported suitable comparative outcomes from a similar study. Following the stepped wedge study design with repeated cross-sectional samples recommendations of Barker et al. [16] and the applied parameters of Bird et al. [5], at least two clusters will be randomised at each step to ensure the nominal 5% significance level. Bird et al. [5] reached statistical significance using ANOVAs and *t* tests over three measures: ED presentations, hospital admissions and bed days. Pre verses post improvement for these three measures was 10%, 25% and 18% respectively. Following consultations with clinical and statistical experts, a conservative effect size across ED and in-patient services utilisation of 9%, a 10% intra-cluster correlation and between-cluster variations of 30% were assumed for the current study. The intervention will be staged in steps, each with at least two clusters/GPs, totalling a minimum of 10 GPs (clusters), who will each recruit 10–12 participants to the study. Allowing for 25% censoring, a sample size of 120 participants will be required, which will provide 80% power to detect a 9% difference (effect size) in service utilisation with statistical significance at the 5% level. The study design incorporates the effects of ‘time’. Sample size calculations were performed using the stepped wedge command in STATA 15 [18].

#### Participant recruitment

The treating GP, enrolled in the study, will select patients based on set eligibility criteria. The GP will discuss the study with individual patients and invite them to provide verbal consent to be contacted by the project manager to further discuss the project. The project team will arrange a time to meet with interested participants to discuss the OPEN ARCH intervention and study requirements, inviting them to provide informed consent for inclusion in the study. Participants who do not wish to provide consent for inclusion in the OPEN ARCH intervention may still access specialist geriatric assessment via the existing Cairns Hospital Outpatient referral pathway, but they will not be offered access to the OPEN ARCH community care coordination or a geriatric assessment at their GP practices.

Participation in this study is voluntary. Patient participants will be informed that they can withdraw at any time without giving a reason and that refusal to participate will not have any impact on their current or future access to geriatric or GP services. If requested by the participant, any information they may have provided will be withdrawn from the study. It is not anticipated that GPs will withdraw their consent to participate in the study. However, should a GP move their practice or retire, their patient participants will also be withdrawn from the study and they will not be invited to participate in any further data collection. Routine geriatric assessments will still be available to all patients via the usual outpatient referral system and any links with services initiated by enablement officers may continue independently of the OPEN ARCH intervention.

#### Inclusion criteria

To be eligible to enter the study, participants must meet the following criteria:

- A community-dwelling older person with chronic conditions and complex care needs, defined as having multiple morbidities or a social situation that requires the attention of multiple healthcare providers or facilities,
  - ○ who is 70 years or older for non-Aboriginal and Torres Strait Islander participants, or 50 years or older for Aboriginal and Torres Strait Islander participants; or
  - ○ who is younger than the previous age criteria but has documented chronic or complex age-related conditions (previously only associated with older persons), such as early-onset dementia or arthritis, or another condition.

#### Exclusion criteria

- Residents of residential aged care facility or nursing homes.
- Currently receiving specialist geriatrician intervention and/or care coordination, such as the Transition Care Program.

### Intervention and comparison

The OPEN ARCH intervention has two components: fast-track specialist geriatric assessment via a community-based referral to a geriatrician in local GP practices; and an enablement service offering coordination and implementation of health and social care services customised to patient need in the community. The OPEN ARCH intervention will be delivered within primary healthcare. Patients who are deemed eligible by GP screening evaluation and meet inclusion criteria will be invited to provide informed consent. 

During the first interview and assessment, the geriatrician will provide an in-depth, standardised comprehensive geriatric assessment (CGA), a multi-dimensional, interdisciplinary process used to quantify an older individual’s medical, psychosocial and functional capabilities, including diagnosis, identification of problems, goal-setting and forming an individualised comprehensive management plan for holistic treatment, rehabilitation, support and follow up [19]. Alternatively, if the participant is unable to attend the assessment at the practice, a geriatrician will offer a home visit. The type and number of referrals to primary allied healthcare professionals and community support services will be determined by individual clinical needs and delivered in the community (GP, allied health practice or community health centre) or at home. Examples of allied health services that will be offered include: occupational therapy, physiotherapy, dietetics and social work. Community social support services such as personal care assistance, meals and domestic assistance will be arranged through My Aged Care and community health providers.

Upon completion of the initial interview and assessment, the geriatrician and enablement officer will meet with GP via face-to-face case conferencing, to complete the CGA with recommendations. The following Medicare Benefits Schedule (MBS) items will be available to GPs to claim: Item Numbers 735, 739 and 743 [20].

Based on the CGA recommendations, the enablement officer will meet with participants to develop individual care plans. The enablement officer, who is an advanced health professional specialising in aged care, will further coordinate the participant’s community care by linking them with existing services. Although a registered clinician, their role is not to provide discipline specific services, but rather to draw on existing public, private and non-government allied health, nursing and social support services available in the community to facilitate care coordination goals. All participants will continue to consult with their GPs for usual medical care.

The baseline (pre-intervention or standard care) level of service provision for older patients includes referrals to the Cairns Hospital Outpatient clinic without multidisciplinary care coordination or referral to teams, such as the Aged Care Assessment Team.

### Blinding

As OPEN ARCH requires active involvement, it would not be possible to blind participants or GPs (clusters), nor enablement officers or the project manager [15]. Participants and GPs will be required to make an informed decision for consent, so they will have full disclosure available prior to the consent. Enablement officers providing clinical care will have full knowledge of the participants’ health status throughout the study period. Similarly, the project manager, who will be required to enter the data, will remain un-blinded throughout the study. However, every attempt will be made to blind outcome assessors in order to reduce information or ascertainment bias [21]. Directly asking outcome assessors which intervention they think was administered will judge the success of blinding.

### Clinical outcomes

#### Primary outcome measures

The primary outcome is service utilisation including: Emergency Department (ED) presentations, hospital admissions, in-patient bed days, allied health and support services utilisation. Hospital and ED data will be extracted from Queensland Health routinely collected computerised patient activity records. The number and type of appointments with the primary care physician and community-based allied health, nursing and social support services will be collated from medical clinic records kept by the engagement officers. A purpose-built data input form will be used to collate health and support service usage.

#### Secondary outcomes measures

Secondary outcomes will comprise: functional status as measured by the Functional Independence Measure [22] and Seniors at Risk (ISAR) screening tool [23]; quality of life (QoL) as measured by EQ-5D-5L [24] and AQOL-8D [25]; and patient experience of the OPEN ARCH intervention collected via the PSQ-18 questionnaire [26]. The secondary outcomes data collection will be shared between the project manager and engagement officers.

### Statistical methods to assess intervention effect

The primary outcome measures, including Emergency Department (ED) presentations, hospital admissions, in-patient bed days, allied health and support services utilisation, will be analysed as panels between each cross-sectional time point of the stepped wedge design, comparing primary outcomes within and between clusters. Each cross-section will include participants undertaking the intervention as well as those receiving standard care (pre-intervention component). In addition to assessing intervention effects, the stepped wedge design can also model the effects of time to determine whether the time of intervention (intervening early versus intervening late) impacts the effectiveness of the intervention. Secondary outcome measures, including functional status, ISAR and QoL measures, will be compared for the pre versus post intervention periods, for each participant, and thus the participants will act as their own control. Participants’ experience of the OPEN ARCH intervention as measured by the Likert scale responses will be analysed using descriptive statistics.

Within-cluster and between-cluster variance, treatment outcomes for intervention versus pre-intervention, across time, will be examined by applying a generalised linear mixed model (GLMM), as recommended for small cluster designed stepped wedge studies [16]. GLMM models can be adjusted for unequal numbers within clusters and cope with non-parametric data distributions. Within the GLMM, primary and secondary outcomes will be assessed against time and intervention status, represented as fixed effects, while study clusters and clients will be represented in the model as random effects [16]. Secondary outcome analyses will include *t* tests (for continuous data), chi-square tests (for proportions) and GLMMs where applicable, to control for demographic differences. All statistical tests will be two-sided and performed using a 5% significance level with no adjustment for multiplicity. Data analysis will be performed using STATA version 15 [18].

### Economic evaluation

The economic research question is whether the OPEN ARCH intervention is cost-effective in reducing hospital utilisation and enhancing health-related quality of life when compared to the standard care. The perspective for the evaluation model will be a healthcare provider in the Australian setting [27].

Costs of the intervention will be incurred during the CGA and management. The CGA and management costs include the average time with each participant of different personnel, such as a GP, community-embedded specialist in geriatric medicine and community-based care coordinator. Costs of consecutive ED presentations, hospital admissions and hospital length of stay will be included. The cost of the pre-intervention care will include healthcare services utilisation before the initial GP consultation and CGA. Trial initiation, patient accrual and pre-screening activities as well as research and evaluation costs will be recorded and reported separately [28, 29].

Resources will be measured on a patient-specific basis obtained from the purpose-built data input form collated by the project manager from routinely collected computerised patient activity records and medical clinic records, following informed consent processes. Utilisation of objective data is known to eliminate patient recall bias and reduce the amount of missing data [30]. The cost of the time will be derived from a log of time spent on direct patient care activities. A top-down approach will be used to value resource use for capital and operating costs. Healthcare resource use data will be valued using Australian Independent Hospital Pricing Authority standard costs [31] reported in 2018 AUS$.

QALYs will be generated using a multi-attribute utility instrument, the EuroQoL 5-Dimension (EQ-5D-5L) scale [24]. This standardised, five-item descriptive system and visual analogue scale measures mobility, self-care, pain, usual activities and anxiety, and contributes to a ‘utility’ score. The AQOL-8D [32] instrument will be used to generate alternative QALYs. The AQOL-8D consists of eight dimensions: physical, including independent living, senses and pain; and psycho-social, including mental health, happiness, self-worth, coping and relationships. Utility weights for the AQOL were generated using the time-trade-off technique and Australian population sample. Due to its robust psychometric properties, the AQOL is recommended for use in epidemiologic studies where health-related QoL and utility data are required from older populations [33]. The AQOL has been used in Australian community older persons and Indigenous Australians studies [25].

The time horizon of the economic analysis will incorporate the lifetime of the cohort, through extrapolation of quality-adjusted survival from the end of the study period. A discount rate of 5% per year will be applied to both costs and outcomes [34]. The difference in costs and QALYs, between the intervention and pre-intervention periods, will be combined into the incremental cost-effectiveness ratio (ICER) with valuations of the willingness to pay for a marginal QALY to estimate the net monetary benefits for a decision to adopt the intervention:

$$ \mathrm{ICER}=\frac{{\mathrm{Cost}}_{\mathrm{Int}}-{\mathrm{Cost}}_{\mathrm{Pre}-\operatorname{int}}}{{\mathrm{QALY}}_{\mathrm{Int}}-{\mathrm{QALY}}_{\mathrm{Pre}-\operatorname{int}}}, $$

where Cost_Int_(Cost_Pre − int_) and QALY_Int_ (QALY_Pre − int_) are the mean cost and QALYs gained in the intervention (pre-intervention) periods, respectively. The maximum willingness to pay for a QALY will be assumed to be AUS$64,000 for the Australian setting [35]. The Markov state transition process will be applied to model participants’ probability of being readmitted to hospital, staying in the community or dying [36]. Sensitivity analysis will be performed to test variations in key parameters including the study perspective, the choice of the comparator and specific cost impacts.

#### Frequency of analyses

All data will be collected at baseline ‘0’ with retrospective collection for 3 months to time point ‘– 1’, following every 3 months of data collection, at time points ‘1’, ‘2’ and ‘3’ (see Fig. 2), except for participants’ experiences, which will be collected at the end of the study period.

### Ethical issues

The human rights and dignity of the participants will be protected in accordance with Australia’s National Health Medical Research Council (NHMRC) guidelines [37]. Participants will not receive any financial inducement to participate. Participants will be provided with contact details of the project team to enable them to address any potential queries or concerns. Aboriginal and Torres Strait Islander participants will be offered the opportunity to have an Aboriginal and Torres Strait Islander Liaison Officer or Health Worker present during their CGA and care plan development. To conform to the Data Protection and Freedom of Information Act, all data will be stored securely and de-identified for the analysis. GPs will have access to the information related to the delivery of the study, including geriatric and enablement officer assessment, planning and progress notes. These documents will be sent securely to the GP to be uploaded to the patient’s medical record. Additionally, as a specialist medical officer, the geriatrician will have access to the GP medical notes into which they will write a summary of geriatric assessment, at the time of client appointment. All aspects of the assessment and patient care planning will be discussed with the GP at the case conference. Participants will be informed of their right to access their own results, and the overall results of the research. No published material will contain patient identifiable information. Any complaints will be systematically recorded and acted upon in accordance with NHMRC guidelines [37].

## Discussion

The OPEN ARCH intervention is based on the geriatric evaluation and management (GEM) model of care. It builds on previous work that explored patient experiences of transitions from the GEM service to primary healthcare [4]. This successful study noted, upon conclusion, that further improvements to geriatric care could be made by offering early intervention and prevention through improved screening and comprehensive assessment in primary care [10].

Thus, this proposed OPEN ARCH study has been designed to address the deficit identified within the original hospital discharge model, through the provision of a community facing preventative care. By providing the care in the community, condition deterioration can be detected earlier and enable timely planning for permanent residential aged care placement, where needed, reducing the likelihood of unexpected hospital admissions from community-residing patients. Furthermore, with fewer hospitalisations, older people are likely to achieve better well-being outcomes if they remain living independently and well supported in the community [2].

The feasibility and sustainability of the OPEN ARCH intervention are ensured by the placement of the intervention within primary care settings and MBS funding available to GPs (Item Numbers 735, 739, 743); utilisation of case conferencing and shared medical records. Further, providing knowledge translation, education and support with system navigation skills to both GPs and practice nurses will ensure a long-term solution to issues related to system functioning and integration. This trial will determine the impact of the OPEN ARCH intervention on the health and quality of life outcomes, health and social services utilisation of older people who have multiple chronic conditions and emerging complex care needs. The trial will involve an economic evaluation to determine whether the model can be considered cost-effective when compared to the standard care.

### Limitations

The study populations are, by definition, at risk of natural functional and cognitive decline that may be marked over the study period. Although the study design applies multiple methods to best capture intervention effects, the benefits in slowing down deterioration may not be successful in some participants due to their frailty. Within the quantitative methods are cross-sectional and case–control (pre–post intervention) analysis, which will assess between and within clusters differences. The pre–post analyses will utilise paired analyses where each participant acts as their own control. These methods plus the inclusion of qualitative surveys should tease out even minor benefits of OPEN ARCH, when presented with accelerated decline. The sample size calculation has allowed attrition, so these factors should allow statistical significant effect size within OPEN ARCH achievable.

## Trial status

In November 2018, the study was 6 months post baseline.

## Additional file

Additional file 1: SPIRIT 2013 Checklist: Recommended items to address in a clinical trial protocol and related documents (DOC 121 kb)

## Abbreviations

CGA: Comprehensive geriatric assessment
CHHHS: Cairns and Hinterland Hospital and Health Service
ED: Emergency Department
GEM: Geriatric evaluation and management
GP: General practitioner
ICER: Incremental cost-effectiveness ratio
NHMRC: National Health Medical Research Council
OPEN ARCH: Older Persons ENablement And Rehabilitation for Complex Health conditions
QALY: Quality adjusted life year
QoL: Quality of life
SPIRIT: Standard Protocol Items: Recommendations for Interventional Trials
TCHHS: Torres and Cape Hospital and Health Service

## References

[1] Australian Institute of Health and Welfare (2014). Australia’s health 2014. Cat no AUS 178.

[2] Beswick AD, Rees K, Dieppe P, Ayis S, Gooberman-Hill R, Horwood J, Ebrahim S (2008). Complex interventions to improve physical function and maintain independent living in elderly people: a systematic review and meta-analysis. Lancet.

[3] National Health and Hospital Reform Commission (2009). A healthier future for all Australians, Final Report of the National Health and Hospital Reform Commission.

[4] Strivens E, Harvey D, Foster M, Quigley R, Wilson M (2015). Analysing sub-acute and primary health care interfaces – research in the elderly. ASPIRE Study.

[5] Bird S, Noronha M, Sinnott H (2010). An integrated care facilitation model improves quality of life and reduces use of hospital resources by patients with chronic obstructive pulmonary disease and chronic heart failure. Aust J Prim Health.

[6] Jack BW, Chetty VK, Anthony D, Greenwald JL, Sanchez GM, Johnson AE, Forsythe SR, O'Donnell JK, Paasche-Orlow MK, Manasseh C (2009). A Reengineered Hospital Discharge Program to Decrease Rehospitalization A Randomized Trial. Ann Intern Med.

[7] Katterl R, Anikeeva O, Butler C, Brown L, Smith B, Bywood P (2012). Potentially avoidable hospitalisations in Australia: Causes for hospitalisations and primary health care interventions. PHCRIS Policy Issue Review.

[8] Arbaje AI, Maron DD, Yu QL, Wendel VI, Tanner E, Boult C, Eubank KJ, Durso SC (2010). The Geriatric Floating Interdisciplinary Transition Team. J Am Geriatr Soc.

[9] Bird SR, Kurowski W, Dickman GK, Kronborg I (2007). Integrated care facilitation for older patients with complex health care needs reduces hospital demand. Aust Health Rev.

[10] Harvey Desley, Foster Michele, Strivens Edward, Quigley Rachel (2017). Improving care coordination for community-dwelling older Australians: a longitudinal qualitative study. Australian Health Review.

[11] Chan AW, Tetzlaff JM, Altman DG, Laupacis A, Gotzsche PC, Krleza-Jeric K, Hrobjartsson A, Mann H, Dickersin K, Berlin JA (2013). SPIRIT 2013 statement: defining standard protocol items for clinical trials. Ann Intern Med.

[12] Chan AW, Tetzlaff JM, Gotzsche PC, Altman DG, Mann H, Berlin JA, Dickersin K, Hrobjartsson A, Schulz KF, Parulekar WR (2013). SPIRIT 2013 explanation and elaboration: guidance for protocols of clinical trials. Bmj.

[13] Queensland Government Statistician’s Office (2016). Queensland statistics.

[14] Census of Population and Housing (ABS Table Builder) [http://www.abs.gov.au/websitedbs/censushome.nsf/home/tablebuilder?opendocument&navpos=240].

[15] Brown CA, Lilford RJ (2006). The stepped wedge trial design: a systematic review. BMC Med Res Methodol.

[16] Barker D, D'Este C, Campbell MJ, McElduff P (2017). Minimum number of clusters and comparison of analysis methods for cross sectional stepped wedge cluster randomised trials with binary outcomes: A simulation study. Trials.

[17] Ivers NM, Halperin IJ, Barnsley J, Grimshaw JM, Shah BR, Tu K, Upshur R, M Z. Allocation techniques for balance at baseline in cluster randomized trials: a methodological review. Trials. 2012;13(120). 10.1186/1745-6215-13-120. 10.1186/1745-6215-13-120 PMC3503622 22853820

[18] StataCorp (2016). Stata: Release 15.1.

[19] Australian and New Zealand Society for Geriatric Medicine (2011). Comprehensive Geriatric Assessment and Community Practice. Position Statement No 8.

[20] Department of Health (2018). Medicare Benefits Schedule (MBS).

[21] Schulz KF, Grimes DA (2002). Blinding in randomised trials: hiding who got what. Lancet.

[22] Functional Indendence Measure (FIM) [http://meteor.aihw.gov.au/content/index.phtml/itemId/495857].

[23] Suijker JJ, Buurman BM, van Rijn M, van Dalen MT, ter Riet G, van Geloven N, de Haan RJ, van Charante EPM, de Rooij SE (2014). A simple validated questionnaire predicted functional decline in community-dwelling older persons: prospective cohort studies. J Clin Epidemiol.

[24] EuroQol Group: EuroQol (1990). A new facility for the measurement of health related quality of life. Health Policy.

[25] Richardson J, Iezzi A, Khan MA, Maxwell A (2014). Validity and reliability of the Assessment of Quality of Life (AQoL)-8D multi-attribute utility instrument. The Patient.

[26] Ware JE, Snyder MK, Wright WR, Davies AR (1983). Defining and measuring patinet satisfaction with medical care. Evaluation and program planning.

[27] National Institute for Health and Clinical Excellence (2013). Guide to the methods of technology appraisal.

[28] Glick HA, Doshi JA, Sonnad SS, Polsky D (2015). Economic Evaluation in Clinical Trials.

[29] Gold MR, Siegel JE, Russell LB, MC W (1996). Cost-Effectiveness in Health and Medicine.

[30] Gray AM, Clarke PM, Wolstenholme JL, Wordsworth S. Applied Methods of Cost-effectiveness Analysis in Healthcare. Oxford University Press; 2010.

[31] Independent Hospital Pricing Authority (2015). Determination of standard costs associated with conducting clinical trials in Australia: Standard List of Clinical Trial Items.

[32] Hawthorne G, Korn S, Richardson J (2013). Population norms for the AQoL derived from the 2007 Australian National Survey of Mental Health and Wellbeing. Aust Nz J Publ Heal.

[33] Osborne RH, Hawthorne G, Lew EA, Gray LC (2003). Quality of life assessment in the community-dwelling elderly: Validation of the Assessment of Quality of Life (AQoL) Instrument and comparison with the SF-36. J Clin Epidemiol.

[34] Department of Health and Ageing Pharmaceutical Benefits Advisory Committee (2008). Guidelines for preparing submissions to the Pharmaceutical Benefits Advisory Committee.

[35] Shiroiwa T, Sung YK, Fukuda T, Lang HC, Bae SC, Tsutani K (2010). International survey on willingness-to-pay (WTP) for one additional QALY gained: what is the threshold of cost effectiveness?. Health Econ.

[36] Sonnenberg FA, Beck JR (1993). Markov models in medical decision making: a practical guide. Medical Decision Making.

[37] National Health and Medical Research Council (2007). National Statement on Ethical Conduct in Human Research.

[38] Hemming K, Taljaard M, Forbes A (2017). Analysis of cluster randomised stepped wedge trials with repeated cross-sectional samples. Trials.

